# Congenital heart diseases with airway stenosis: a predictive nomogram to risk-stratify patients without airway intervention

**DOI:** 10.1186/s12887-023-04160-5

**Published:** 2023-07-12

**Authors:** Qiyu He, Yuze Liu, Zheng Dou, Kai Ma, Shoujun Li

**Affiliations:** Pediatric Cardiac Surgery Centre, National Centre for Cardiovascular Diseases, State Key Laboratory of Cardiovascular Disease, Fuwai Hospital, Chinese Academy of Medical Sciences, Peking Union Medical College, Beijing, 100037 China

**Keywords:** Congenital heart diseases, Airway stenosis, Mechanical ventilation, Nomogram, Predictive model

## Abstract

**Background:**

This study focused on congenital heart disease (CHD) patients complicated with airway stenosis (AS) without airway intervention and aimed to identify the patients with potential risks.

**Methods:**

Patients diagnosed with CHD and AS were enrolled in this retrospective study. The primary outcome was defined as a postoperative mechanical ventilation duration of more than two weeks. We constructed a prediction model to predict the risk of prolonged mechanical ventilation (PMV).

**Results:**

A total of 185 patients diagnosed with CHD and AS in Fuwai Hospital from July 2009 to December 2022 were included in the study. Weight at CHD surgery, cardiopulmonary bypass (CPB) duration, complex CHD and comorbid tracheobronchomalacia were identified as risk factors and included in the model. The ROC curve showed a good distinguishing ability, with an AUC of 0.847 (95% CI: 0.786–0.908). According to the optimal cut-off value of the ROC curve, patients were divided into high- and low-risk groups, and the subsequent analysis showed significant differences in peri-operative characteristics and in-hospital deaths.

**Conclusions:**

With the predictive model, several factors could be used to assess the risky patients with PMV. More attention should be paid to these patients by early identification and routine surveillance.

**Supplementary Information:**

The online version contains supplementary material available at 10.1186/s12887-023-04160-5.

## Background

Congenital heart disease (CHD) complicated with airway anomaly, a rare combination of lesions, approximately accounts for 3–4% of patients [1]. Tracheobronchial malacia, tracheal stenosis, and tracheoesophageal fistulas are the major presentations of the anomalies, and surgical intervention may be needed occasionally [2, 3]. Airway stenosis (AS) is the major subtype and 50–70% of the patients are reported to have a concomitant cardiac anomaly [4–6].

Depending on the various presenting symptoms, different treatment strategies may be adopted. For severe conditions, surgical intervention may be needed. However, for AS patients with moderate respiratory symptoms, conservative management may be preferred. Cheng et al. indicated that compared to normal children, tracheal growth and tracheal diameter enlargement seem to be faster in congenital tracheal stenosis (CTS) children, especially after infancy [7]. In a large retrospective analysis of the Society of Thoracic Surgeons Congenital Heart Surgery Database (STS-CHSD), 6861 (3.4%) patients were complicated with airway anomalies and only 428 (0.2%) patients underwent tracheal operations during the same hospitalization [8].

Most of the studies focused on CHD patients who underwent tracheal surgery, so the data on the conservative group are scarce [9, 10]. As we mentioned, most CHD patients with tracheal anomalies may not require tracheal intervention, thus, studies exploring these populations are warranted to better understand the outcomes and prognosis. Consequently, in this study, we aim to retrospectively investigate the CHD patients who underwent conservative management of AS in our center, to provide novel insight to AS management.

## Methods

### Patient population and management

Patients with the diagnoses of CHD complicated with AS in Fuwai Hospital were reviewed from July 2009 to December 2022. For patients diagnosed with AS and CHD, computered tomography (CT) scan or fiberoptic bronchoscopy would be prescribed. After adequate multidisciplinary team (MDT) consultation and preoperative evaluation by cardiac surgeons, cardiologists, echocardiologists, and radiologists, patients with stenotic airway segment detected by fiberoptic bronchoscopy or CT scan and significant respiratory symptoms would undergo tracheal surgery, such as slide tracheoplasty. However, in patients with AS reported by bronchoscopy or CT but mild or asymptomatic respiratory symptoms, conservative treatment on AS would be preferred. Consequently, we enrolled patients who met the following criteria: diagnosis of CHD, AS determined by fiberoptic bronchoscopy or CT scan, no significant respiratory symptom, and no tracheal surgery during hospitalization.

### Definitions

The Society of Thoracic Surgeons- European Association for Cardiothoracic Surgery (STAT) mortality category was selected to risk-stratify the CHD procedures. Complex CHD was defined as CHDs excluding simple shunts (atrial septal defect, ventricular septal defect and patent ductus arteriosus), pulmonary artery slings and vascular rings [11]. The primary outcome was defined as prolonged mechanical ventilation (PMV). According to previous reports and our clinical experience, PMV was defined as postoperative mechanical ventilation for more than 14 days [12–14].

### Statistical analysis

Descriptive analysis of baseline data was presented by median (interquartile range, IQR) for continuous variables, or frequency (percentage) for categorical variables. Wilcoxon rank sum test were used to compare the continuous data and the chi-square test or Fisher exact test was used to analyze categorical data. Variables with missing values were detected and processed by the Mice package of R. The imputation methods were selected based on the type of variables that continuous data were imputed using Predictive Mean Matching, and categorical data were imputed using Logistic Regression Imputation. The imputed dataset was compared with the original dataset to verify the bias generated in the process. The imputed dataset was used for the following statistical analysis.

Logistic regression was used to assess the association between perioperative indicators and prognosis. Variables with potential predictive power were first included in the univariate logistic regression analysis, and those with P < 0.1 were further included in the multivariate logistic regression analysis.

We tested the accuracy of the model with the Hosmer-Lemeshow test and presented it with calibration plots. The receiver operating characteristic (ROC) curve was plotted to determine the model’s ability to accurately identify PMV in children with CHD undergoing conservative treatment of AS. We also identified the net benefit of the model by decision curve analysis (DCA). Finally, a nomogram was adopted to visualize the model.

Due to the lack of an external validation cohort, we internally validated the model using machine learning method. Cross-validation was selected to examine the accuracy and analyze the overfitting of the predictive model. After randomly divided all the data in the original cohort into k groups, (k-1) of them were taken as the training cohort for constructing the model, and the remaining 1 group was treated as the validation cohort (k-fold cross-validation). The model constructed from the training cohort was used to predict the outcome of the validation cohort. To reduce the influence of random grouping, the above process was repeated 200 times. The other method we used for internal validation was bootstrap validation. The training cohort was constructed by sampling (with replacement) from the original cohort. Although the probability of each patient being selected was the same, the training cohort differed significantly from the original cohort due to the use of sampling with replacement. Similarly, the process was repeated 200 times. We used these two methods to obtain the corrected area under curve (AUC) values and 95% confidence intervals for model correction, respectively.

Based on the results of logistic regression, the possible risk score for each patient was calculated, and the patients were divided into high-risk and low-risk groups according to the optimal cutoff value of the ROC curve. We further compared the differences in other postoperative indicators between the two groups, including postoperative hospital stay, ICU stay, reintubation, ECMO implication, and in-hospital death.

All the statistical analyses were performed using R-studio (version 4.2.2), P < 0.05 was considered to be statistical significance.

## Results

### Baseline characteristics

This retrospective study comprised 221 patients diagnosed with CHD and AS, of which 36 patients underwent airway surgery (15 with slide tracheoplasty, 21 with tracheobronchial external suspension) were excluded, and a total of 185 patients were enrolled in the final cohort. Based on the primary outcome set for the model, 49 patients (26.5%) were allocated to the PMV group. The medium age for the whole group was 0.7 years old (IQR, 0.39–1.54), with 44.0% of the patients being female. Regarding different CHD procedures, the STAT category was adopted for the risk stratification and differences were mainly observed in STAT 1, STAT 3, STAT 4, and STAT 5 categories. 22 patients (59%) in the PMV group were comorbid with tracheobronchomalacia. According to the grouping, the perioperative outcomes, including CPB, ACC, ICU stay, postoperative length of stay, reintubation, ECMO implication, postoperative tracheotomy, ventilator-associated pneumonia, pneumothorax, and in-hospital death were significantly different between the two groups (P < 0.05). There were ten in-hospital deaths, eight of which belonged to the PMV group. Detailed information of baseline characteristics of the whole cohort was shown in Table 1 and Supplementary Table 1.

**Table 1:** Baseline characteristics

	Overall (N = 185)	Mechanical ventilation duration		P-value
		≤ 2 weeks (N = 136)	> 2 weeks (N = 49)	
Gestational age, weeks	39.00 (38.00, 40.00)	39.00 (38.00, 40.00)	38.00 (37.00, 40.00)	0.4
Weight at birth, kg	3.10 (2.70, 3.50)	3.15 (2.70, 3.50)	3.10 (2.80, 3.40)	0.7
Age at CHD surgery, years	0.70 (0.39, 1.54)	0.74 (0.44, 1.73)	0.58 (0.25, 0.93)	0.004
Weight at CHD surgery, kg	7.2 (5.5, 10.0)	7.5 (6.0, 10.7)	6.1 (5.0, 7.7)	< 0.001
Height at CHD surgery, cm	66 (61, 78)	70 (63, 80)	63 (57, 69)	< 0.001
CPB duration, min	92 (56, 133)	72 (38, 115)	126 (96, 182)	< 0.001
ACC duration, min	48 (6, 80)	42 (0, 70)	77 (52, 114)	< 0.001
Mechanical ventilation, hours	36 (7, 375)	20 (5, 52)	777 (500, 1,323)	< 0.001
Postoperative ICU-stay, days	6 (2, 26)	4 (2, 8)	45 (33, 66)	< 0.001
Postoperative hospital-stay, days	20 (13, 40)	15 (11, 20)	53 (41, 82)	< 0.001
Gender				0.6
Male	104 (56%)	75 (55%)	29 (59%)	
Female	81 (44%)	61 (45%)	20 (41%)	
STAT category				< 0.001
STAT1	73 (39%)	67 (49%)	6 (12%)	
STAT2	55 (30%)	39 (29%)	16 (33%)	
STAT3	36 (19%)	21 (15%)	15 (31%)	
STAT4	16 (8.6%)	9 (6.6%)	7 (14%)	
STAT5	5 (2.7%)	0 (0%)	5 (10%)	
Complex CHD	119 (64%)	77 (57%)	42 (86%)	< 0.001
Tracheobronchomalacia	56 (30%)	27 (20%)	29 (59%)	< 0.001
Tracheobronchial compression	57 (31%)	46 (34%)	11 (22%)	0.14
Congenital tracheal stenosis	18 (9.7%)	17 (12%)	1 (2.0%)	0.046
Stenotic segment				0.057
Tracheal	74 (40%)	60 (44%)	14 (29%)	
Bronchus	111 (60%)	76 (56%)	35 (71%)	
Reintubation	26 (14%)	9 (6.6%)	17 (35%)	< 0.001
ECMO use	11 (5.9%)	0 (0%)	11 (22%)	< 0.001
Postoperative tracheotomy	25 (14%)	2 (1.5%)	23 (47%)	< 0.001
Ventilator-associated pneumonia	40 (22%)	10 (7.4%)	30 (61%)	< 0.001
Pneumothorax	8 (4.3%)	2 (1.5%)	6 (12%)	0.005
In-hospital deaths	10 (5.4%)	2 (1.5%)	8 (16%)	< 0.001

Value are presented with median (IQR) for continuous variables, number (percentage) for categorical variables. CHD: Congenital heart disease; CPB: Cardiopulmonary bypass; ACC: Aortic cross-clamping; ICU: Intensive care unit; ECMO: Extracorporeal membrane oxygenation. Complex CHD excludes simple shunts (atrial septal defect, ventricular septal defect, patent ductus arteriosus), vascular rings, and pulmonary artery slings.

### Data imputation

Several variables in the original dataset contained various degrees of missing data, including gestational age (18/185, 9.7%), birth weight (21/185, 11.4%), weight at CHD surgery (1/185, 0.5%), height at CHD surgery (4/185, 2.2%), cardiopulmonary bypass (CPB) duration (1/185, 0.5%) and aortic cross clamp (ACC) duration (1/185, 0.5%). Multiple imputation methods were utilized to fill in the missing values. The imputed dataset was compared with the original dataset, and the results showed that the imputation did not cause any significant bias (Supplementary Table 2).

### Predictive model

**Fig. 1:**
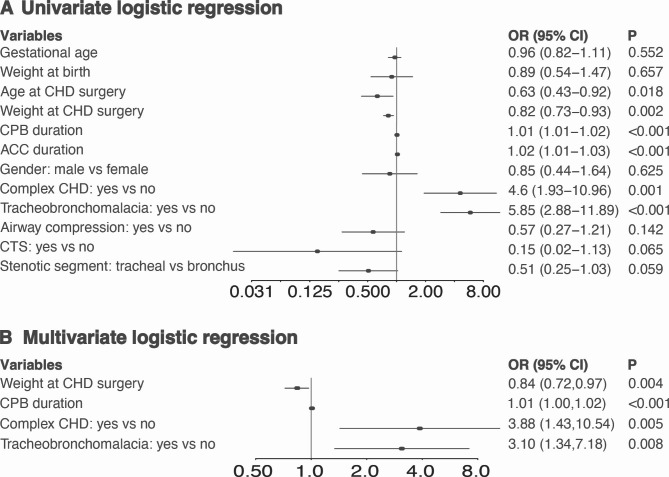
Factors associated with PMV. (**A**) Univariate logistic regression. Factors with predictive potential were included in the univariate logistic regression, and the result was presented with a forest plot. (**B**) Multivariate logistic regression. Four factors were included in the predictive model and presented with a forest plot, including weight at CHD surgery, CPB duration, complex CHD, and tracheobronchomalacia. P < 0.05 was considered to be statistically significant

**Fig. 2:**
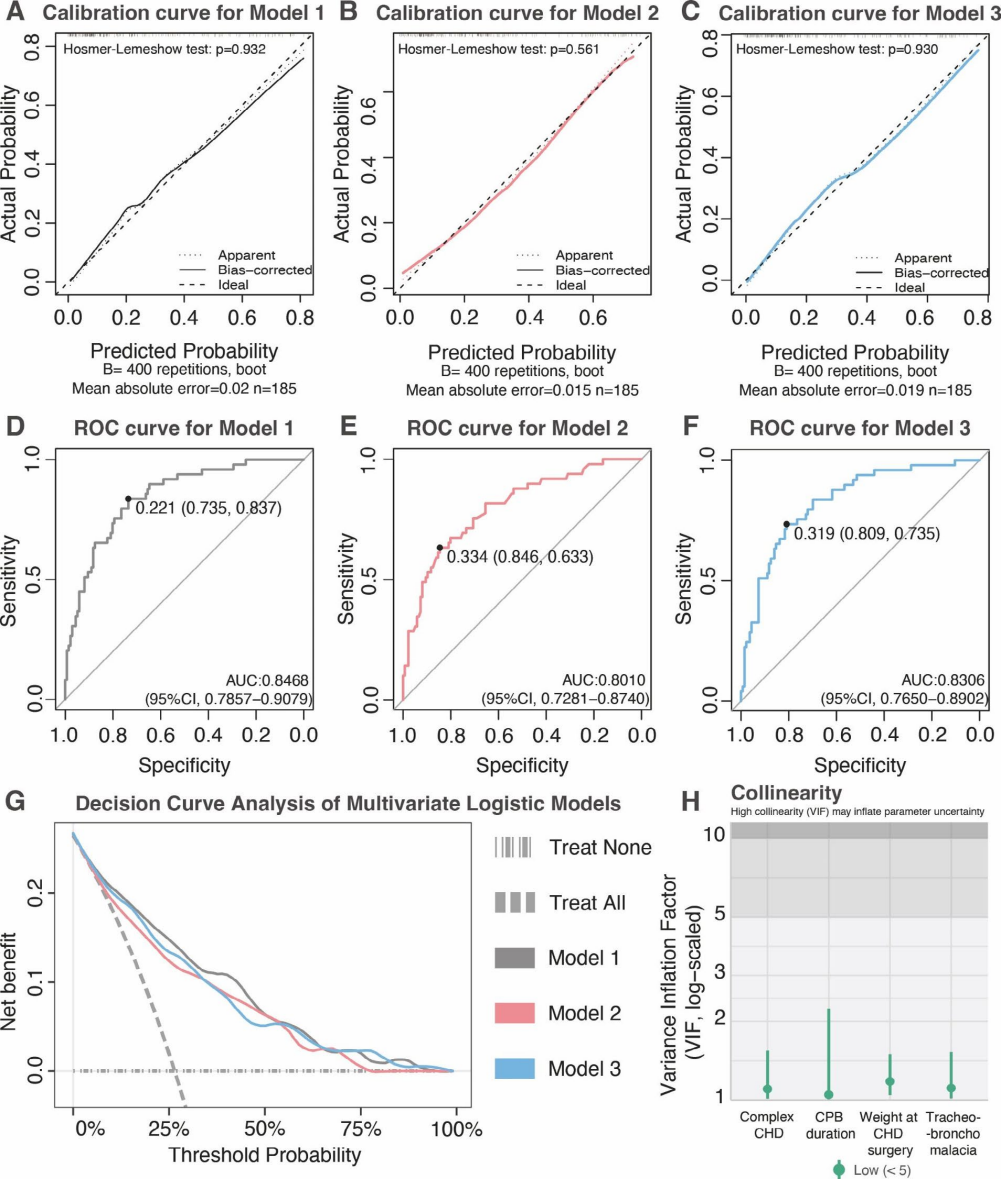
The diagnosis of the predictive model. (**A-C**) Calibration curves for logistic regression models. The predicted probability of PMV was presented on the x-axis, and the actual probability was presented on the y-axis. All three models passed the Hosmer-Lemeshow test (P > 0.05); (**D-F**) ROC curves for logistic regression models. The ROC curves and optimal cut-off values for Model 1 (grey), Model 2 (red), and Model 3 (blue) were plotted; (**G**) Decision curve analysis for PMV prediction. We preferred the prediction model with higher net benefit values over a larger threshold probability range. All three prediction models had higher net benefits than the control line (treat all and treat none) over the full range of threshold probabilities (**H**) Diagnosis of multicollinearity of Model 1. The result showed no multicollinearity (Variance Inflation Factor < 5). Variables: Model 1: weight at CHD surgery, CPB duration, complex CHD and comorbid tracheobronchomalacia; Model 2: weight at CHD surgery, complex CHD and comorbid tracheobronchomalacia; Model 3: weight at CHD surgery, CPB duration and comorbid tracheobronchomalacia

Twelve variables were included in the predictive model with univariate logistics regression analysis (Fig. 1A). Multivariate logistic regression indicated four independent risk factors for PMV, including weight at CHD surgery, CPB duration, complex CHD and comorbid tracheobronchomalacia (Fig. 1B). Since both complex CHD and CPB duration represented the complexity of the surgical procedure, we built additional two different logistic regression models (Model 2 [weight at CHD surgery + complex CHD + comorbid tracheobronchomalacia] and Model 3 [weight at CHD surgery + CPB duration + comorbid tracheobronchomalacia]) to explore the contribution of two variables. The likelihood ratio test for the regression model showed statistically significant differences between Model 1, Model 2 and Model 3 (Model 1 vs. Model 2: P < 0.001; Model 1 vs. Model 3 P = 0.005). As the calibration plot indicated, the predicted probability overlapped with the actual probability in all three models, showing the consistency between regression models and actual observations (Fig. 2A-C). The Hosmer-Lemeshow test also showed no statistically significant difference between the predicted and actual values (Model 1: ${\mathcal{X}}^{2}$=3.041, P=0.932; Model 2: ${\mathcal{X}}^{2}$=6.773, P = 0.561; Model 3: ${\mathcal{X}}^{2}$=3.061, P = 0.931). According to the ROC analysis, the AUC for Model 1, Model 2 and Model 3 were 0.847 (95% CI: 0.786–0.908), 0.801 (95% CI: 0.729–0.874), 0.831 (95% CI: 0.765–0.890), and the recommended cut-off values for each model were 0.221, 0.334 and 0.319, respectively (Fig. 2D-F). Next, we examined the clinical performance of these three regression models with decision curve analysis (Fig. 2G). Compared to the control line, Model 1, Model 2 and Model 3 resulted in high net benefit values in a wide range of threshold probabilities, especially Model 1.

**Fig. 3:**
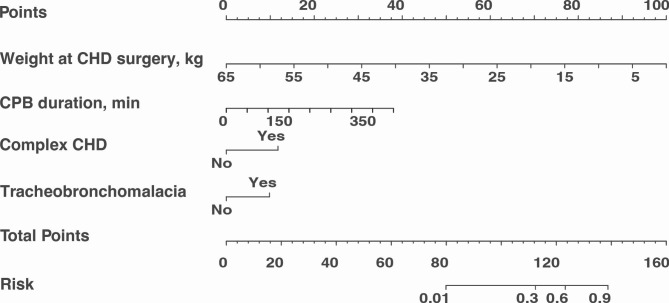
The nomogram for predicting the risk of PMV. The corresponding total score was calculated based on the value of each variable. The corresponding risk of PMV was based on the total score

After evaluating the models for prediction accuracy and clinical implication, Model 1 was selected as the final prediction model. All variables in Model 1 were included in the multicollinearity test, and no multicollinearity was observed (Fig. 2H). A nomogram based on logistic regression was plotted to visualize the predictive model (Fig. 3). The 3, 5, and 10-fold cross-validation and the bootstrap validation indicated that the predictive model has good accuracy and no obvious over-interpretation (Supplementary Table 3).

The values (or classifications) of these perioperative indicators were used to score each patient’s risk and thus the probability of PMV was calculated. According to the cut-off value of the ROC curve, patients were divided into high-risk and low-risk groups. In addition to the duration of mechanical ventilation, other important postoperative factors differed between groups based on predictive models, such as post-operative length of stay, ICU duration, and in-hospital deaths (Table 2).

**Table 2:** Postoperative features of low-/high-risk groups

Variables	Low-risk group (N = 108)	High-risk group (N = 77)	P-value
Mechanical ventilation, hours	16 (5, 36)	375 (70, 776)	< 0.001***
Postoperative ICU-stay, days	3 (1, 6)	24 (11, 46)	< 0.001***
Postoperative hospital-stay, days	14 (10, 20)	35 (20, 56)	< 0.001***
Reintubation	6 (5.6%)	20 (26%)	< 0.001***
ECMO use	1 (0.9%)	10 (13%)	< 0.001***
Postoperative tracheotomy	7 (6.5%)	18 (23%)	< 0.001***
Ventilator-associated pneumonia	11 (10%)	29 (38%)	< 0.001***
Pneumothorax	5 (4.6%)	3 (3.9%)	0.9
In-hospital deaths	1 (0.9%)	9 (12%)	0.002**

Values are presented with median (IQR) for continuous variables, number (percentage) for categorical variables. ICU: Intensive care unit; ECMO: Extracorporeal membrane oxygenation.

## Discussion

This is the first study focusing on CHD patients with AS who underwent conservative airway treatment. Plenty of studies have investigated the surgical outcomes of CHD patients who underwent airway surgery, while the data on AS without intervention are scarce. Riggs and colleagues retrospectively reviewed the associated airway anomaly in pediatric patients who underwent heart surgery from the STS-CHSD, and this study provided insights in patients with AS but without surgical intervention that the operative mortality of 5.9%, major morbidity of 21.2%, and postoperative tracheotomy of 5.6%, respectively [8]. As a result, we sought to take a deep insight into this population and provided more information on their perioperative characteristics, by retrospectively reviewing the patients who met the criteria from 2009 to 2022 in the National Center for Cardiovascular Center of China.

The mechanical ventilation duration is an important perioperative indicator for children undergoing cardiac surgery, especially children with airway anomalies, yet there was no consensus definition of PMV in children. According to a systemic review, this definition varied from 48 h to 6 months [15]. Polito et al. retrospectively investigated the mechanical ventilation duration of patients after complex congenital cardiac surgery, and the cohort comprised 362 patients, of whom 41 (11%) required mechanical ventilation for ≥ 7 days (median ventilation duration for 362 patients: 1.5 days, range: 0–7 days) [13]. However, the mechanical ventilation duration of CHD patients was longer when complicated with airway anomaly. McMahon et al. reported that pediatric patients with CHD and bronchopulmonary dysplasia had a median postoperative mechanical ventilation duration of 15 days (range: 1-141) [16]. For patients with CHD and CTS, the median ventilation duration was 9 days (IQR, 5-20.75) [11]. Given that the coexisting airway stenosis may extend the mechanical ventilation duration compared to patients with CHD only, we established the standard of PMV at a higher level (≥ 14 days). We also referenced results from PICU, where patients with various conditions, not limited to cardiovascular disease. A cohort enrolled children in PICU showed the incidence of PMV (≥ 14 days) is 33.2%, identifying an elevated rate of extubation failure, increased hospitalization costs, and higher mortality after 1-month discharge in patients who received PMV [17].

The PMV is associated with ICU stay duration, re-intubation, perioperative complications, and increased mortality [18, 19]. There were several research focusing on the predictive factors for mechanical ventilation after pediatric cardiac surgery. Gaies et al. developed a model to predict the mechanical ventilation duration after pediatric cardiac surgery, revealing that age, prematurity, extracardiac/genetic anomalies, underweight, preoperative mechanical ventilation, higher STAT category (STAT 4 and 5), and cardiopulmonary bypass duration were the independent predictors [20]. According to a multicenter study performed by Gupta et al., the odds of mechanical ventilation after cardiac surgery were associated with patient characteristics, surgical risk category, and cardiac center volume [21]. Similarly, Maisat et al. identified risk factors for postoperative mechanical ventilation for pediatric pulmonary vein stenosis, including male sex, low body weight, preoperative oxygen supplement, high PVS severity score, intraoperative red blood cell transfusion low preintervention PaO2/ FIO2 ratio, and high preintervention right ventricular systolic pressure [22]. According to our study, four predictive factors were identified when focusing on children with CHD and AS: weight at CHD surgery, CPB duration, complex CHD, and comorbid tracheobronchomalacia. CHD, especially great vessel anomalies can cause airway compression, contributing to tracheobronchomalacia [23]. According to Chen et al., the combination of CHD and tracheobronchomalacia was associated with PMV, ICU-stay, hospital-stay, and mortality [24]. For a subset of patients with airway compression or tracheobronchomalacia, invasive airway intervention may be waived after the relief of compression or stenosis by cardiac surgery.

Patients included in our cohort had a relatively young age (median 0.70, IQR 0.39–1.54), which could be attributed to the growth of children’s airways. As their bodies grow, the length, diameter, and cross-sectional area of the airway increase, and this growth process continues into adulthood [25]. In comparison to the trachea in adults, the size of the trachea during infancy is approximately 50%, 36%, and 15% of the length, diameter, and cross-sectional area, respectively [26]. The growth of the trachea has been observed in patients with CTS as well, with the diameter approaching normal values by the age of nine [7].

Our study developed a nomogram-based prediction model and the corresponding risk scores could be calculated. The predictive model identified patients who had a high risk for PMV, and those patients may tend to have worse postoperative outcomes, including postoperative ICU-stay and hospital-stay, reintubation, ECMO use, postoperative tracheotomy, and in-hospital death (Table 2). Therefore, early identification of this group is essential that more attention should be paid on intensive care of these patients in the perioperative period, and more comprehensive as well as routine surveillance of these patients should be set up after discharge to further improve the prognosis.

Efforts were made to shorten the mechanical ventilation duration. According to a randomized clinical trial by Blackwood et al., compared to usual care in the pediatric ICU, a sedation and ventilator liberation protocol including assessment of sedation levels, spontaneous breathing trials, and non-invasive ventilator resulted in a statistically significant reduction in time to the first successful extubation [27]. Tracheotomy during ventilator adoption is also considered to be beneficial in reducing mechanical ventilation. Each pediatric intensive care unit has different options for the timing of tracheostomy [28]. A recent review suggested that performing a tracheotomy early may improve important medical outcomes [29]. Other management included specific body positions for receiving mechanical ventilation and intraoperative protective ventilation [30, 31]. Strategies for reducing the duration of mechanical ventilation in patients with congenital heart disease combined with AS are still under discussion. However, one thing is certain, early identification of those at risk for PMV is necessary.

### Limitations

This study has several limitations to be addressed. First, as the present study was a single-center retrospective study, validation of an external cohort was lacking. The model’s predictive power should be tested in an external cohort by calculating each patient’s risk score and comparing it to their actual outcomes. Although we performed adequate internal validation to assess whether the model was overfitted, we were still unable to confirm whether the model was applicable to all patients. A subsequent prospective study may serve as an external validation cohort to evaluate the external applicability of this model. Second, patients included in the study suffered not only from AS but also various cardiac malformations, the influence of cardiac surgery could not be ignored, even if we tried to reduce this imbalance, the final results might still be influenced. Finally, our study included all the patients who met the inclusion criteria between July 2009 and January 2022, it is possible that the sample size was inadequate, albeit to the rarity of CHD complicated with AS [32]. The optimal sample size for the predictive model was calculated according to the 4-step sample size calculation method proposed by Riley et al. [32]. Since no similar prediction model was available for our reference, the parameters we set when using the 4-step method were based on the model we built, the calculation results could only be used for model evaluation and subsequent model refinement. With an outcome incidence of 0.265, an AUC value of 0.847, and 4 variables planned to be included as candidates, a sample size of 300 was calculated using the *pmsampsize* package of R, and 80 of these outcome events should be observed.

## Conclusions

We provided a predictive nomogram model to predict the postoperative PMV in patients with CHD and AS who underwent non-surgical airway intervention. The nomogram we plotted could be used to identify those patients at risk. These patients might be benefited from early identification, with more intensive monitoring and extra airway management.

## Electronic supplementary material

Below is the link to the electronic supplementary material.

Supplementary Material 1

Supplementary Material 2

Supplementary Material 3

## Data Availability

The data used in this current study are available from the corresponding author on reasonable request.

## List of abbreviations

ACC: Aortic cross clamp
AIC: Akaike information criterion
AS: Airway stenosis
AUC: Area under curve
CHD: Congenital heart diseases
CPB: Cardiopulmonary bypass
CT: Computered tomography
CTS: Congenital tracheal stenosis
DCA: Decision curve analysis
ECMO: Extracorporeal membranous oxygenation
ICU: Intensive care unit
IQR: Interquartile range
MDT: Multidisciplinary treatment
ROC: Receiver operating characteristic
STAT: Society of Thoracic Surgeons- European Association for Cardiothoracic Surgery
STS-CHSD: Society of Thoracic Surgeons Congenital Heart Surgery Database
VIF: Variance inflation factor
