# Supplementary material for: Congenital heart diseases with airway stenosis: a predictive nomogram to risk-stratify patients without airway intervention
Source: BMC Pediatr. 2023 Jul 12;23:351. doi: 10.1186/s12887-023-04160-5 (PMC10337114; doi:10.1186/s12887-023-04160-5)
Supplement: Supplementary file 1 — Supplementary Material 1 [file 12887_2023_4160_MOESM1_ESM.docx]

**Supplementary Table 1.** Major cardiac anomalies of patients in the cohort.

| **Cardiac anomalies** | **Number (Proportion)** |
| --- | --- |
| Anomalous aortic origin of the coronary artery | 4 (2.2%) |
| Aortic aneurysm | 1 (0.5%) |
| Aortic stenosis | 1 (0.5%) |
| Atrial septal defect | 4 (2.2%) |
| Atrial aneurysm | 1 (0.5%) |
| Atrioventricular septal defect | 7 (3.8%) |
| Coarctation of aorta | 16 (8.6%) |
| Cor triatrium | 1 (0.5%) |
| Double outlet right ventricle | 12 (6.5%) |
| Double aortic arch | 12 (6.5%) |
| Hypertrophic obstructive cardiomyopathy | 1 (0.5%) |
| Interrupted aortic arch | 2 (1.1%) |
| Mitral valve regurgitation | 2 (1.1%) |
| Partial anomalous pulmonary venous connection | 2 (1.1%) |
| Pulmonary artery sling | 14 (7.6%) |
| Patent ductus arteriosus | 8 (4.3%) |
| Pulmonary atresia | 5 (2.7%) |
| Pulmonary valve stenosis | 2 (1.1%) |
| Pulmonary vein stenosis | 2 (1.1%) |
| Right aortic arch | 4 (2.2%) |
| Single ventricle | 3 (1.6%) |
| Transposition of the great arteries | 12 (6.5%) |
| Tetralogy of Fallot | 19 (10%) |
| Tricuspid valve regurgitation | 1 (0.5%) |
| Truncus arteriosus | 1 (0.5%) |
| Vascular ring | 2 (1.1%) |
| Ventricular septal defect | 46 (25%) |

Some patients had more than one cardiac defect, only the major cardiac anomaly was listed in the table.
