# Supplementary material for: Congenital heart diseases with airway stenosis: a predictive nomogram to risk-stratify patients without airway intervention
Source: BMC Pediatr. 2023 Jul 12;23:351. doi: 10.1186/s12887-023-04160-5 (PMC10337114; doi:10.1186/s12887-023-04160-5)
Supplement: Supplementary file 2 — Supplementary Material 2 [file 12887_2023_4160_MOESM2_ESM.docx]

**Supplementary Table 3.** Internal validation result.

| **Method** | **Iterations** | **Adjusted AUC (95% CI)** |
| --- | --- | --- |
| 3-fold cross validation | 200 | 0.820 (0.813, 0.827) |
| 5-fold cross validation | 200 | 0.830 (0.820, 0.840) |
| 10-fold cross validation | 200 | 0.829 (0.814, 0.844) |
| Bootstrap validation | 200 | 0.844 (0.840, 0.848) |
