# Supplementary material for: Congenital heart diseases with airway stenosis: a predictive nomogram to risk-stratify patients without airway intervention
Source: BMC Pediatr. 2023 Jul 12;23:351. doi: 10.1186/s12887-023-04160-5 (PMC10337114; doi:10.1186/s12887-023-04160-5)
Supplement: Supplementary file 3 — Supplementary Material 3 [file 12887_2023_4160_MOESM3_ESM.docx]

**Supplementary Table 2.** Comparison of variables with missing values between original dataset and imputed dataset.

|  | **Before imputation (N=185)** | **After imputation (N=185)** | **P-value** |
| --- | --- | --- | --- |
| **Gestational age, weeks** | 39.00 (38.00, 40.00) | 39.00 (38.00, 40.00) | 0.9 |
| **Weight at birth, kg** | 3.10 (2.70, 3.50) | 3.10 (2.70, 3.40) | 0.6 |
| **Weight at CHD surgery, kg** | 7.2 (5.5, 10.0) | 7.2 (5.5, 10.0) | >0.9 |
| **Height at CHD surgery, cm** | 66 (61, 78) | 66 (61, 78) | >0.9 |
| **CPB duration, min** | 92 (56, 133) | 91 (54, 133) | >0.9 |
| **ACC duration, min** | 48 (6, 80) | 48 (6, 80) | >0.9 |

Median (IQR) for continuous variables.

CHD: Congenital heart disease; CPB: Cardiopulmonary bypass; ACC: Aortic cross clamp.
